# Variant type and position predict two distinct limb phenotypes in patients with GLI3-mediated polydactyly syndromes

**DOI:** 10.1136/jmedgenet-2020-106948

**Published:** 2020-06-26

**Authors:** Martijn Baas, Elise Bette Burger, Ans MW van den Ouweland, Steven ER Hovius, Annelies de Klein, Christianne A van Nieuwenhoven, Robert Jan H Galjaard

**Affiliations:** 1 Plastic, Reconstructive and Hand Surgery, Erasmus MC, Rotterdam, The Netherlands; 2 Clinical Genetics, Erasmus MC, Rotterdam, Zuid-Holland, The Netherlands; 3 Plastic, Reconstructive and Hand Surgery, Radboud University Nijmegen, Nijmegen, Gelderland, The Netherlands; 4 Hand and Wrist Centre, Xpert Clinic, Eindhoven, The Netherlands

**Keywords:** developmental, molecular genetics, clinical genetics, genetic screening/counselling

## Abstract

**Introduction:**

Pathogenic DNA variants in the GLI-Kruppel family member 3 (*GLI3)* gene are known to cause multiple syndromes: for example, Greig syndrome, preaxial polydactyly-type 4 (PPD4) and Pallister-Hall syndrome. Out of these, Pallister-Hall is a different entity, but the distinction between Greig syndrome and PPD4 is less evident. Using latent class analysis (LCA), our study aimed to investigate the correlation between reported limb anomalies and the reported *GLI3* variants in these GLI3-mediated polydactyly syndromes. We identified two subclasses of limb anomalies that relate to the underlying variant.

**Methods:**

Both local and published cases were included for analysis. The presence of individual limb phenotypes was dichotomised and an exploratory LCA was performed. Distribution of phenotypes and genotypes over the classes were explored and subsequently the key predictors of latent class membership were correlated to the different clustered genotypes.

**Results:**

297 cases were identified with 127 different variants in the *GLI3* gene. A two-class model was fitted revealing two subgroups of patients with anterior versus posterior anomalies. Posterior anomalies were observed in cases with truncating variants in the activator domain (postaxial polydactyly; hand, OR: 12.7; foot, OR: 33.9). Multivariate analysis supports these results (Beta: 1.467, p=0.013 and Beta: 2.548, p<0.001, respectively). Corpus callosum agenesis was significantly correlated to these variants (OR: 8.8, p<0.001).

**Conclusion:**

There are two distinct phenotypes within the GLI3-mediated polydactyly population: anteriorly and posteriorly orientated. Variants that likely produce haploinsufficiency are associated with anterior phenotypes. Posterior phenotypes are associated with truncating variants in the activator domain. Patients with these truncating variants have a greater risk for corpus callosum anomalies.

## Introduction

GLI-Kruppel family member 3 (*GLI3*) encodes for a zinc finger transcription factor which plays a key role in the sonic hedgehog (SHH) signalling pathway essential in both limb and craniofacial development.^1 2^ In hand development, SHH is expressed in the zone of polarising activity (ZPA) on the posterior side of the handplate. The ZPA expresses SHH, creating a gradient of SHH from the posterior to the anterior side of the handplate. In the presence of SHH, full length GLI3-protein is produced (GLI3A), whereas absence of SHH causes cleavage of GLI3 into its repressor form (GLI3R).^3 4^ Abnormal expression of this SHH/GLI3R gradient can cause both preaxial and postaxial polydactyly.^2^

Concordantly, pathogenic DNA variants in the *GLI3* gene are known to cause multiple syndromes with craniofacial and limb involvement, such as: acrocallosal syndrome^5^ (OMIM: 200990), Greig cephalopolysyndactyly syndrome^6^ (OMIM: 175700) and Pallister-Hall syndrome^7^ (OMIM: 146510). Also, in non-syndromic polydactyly, such as preaxial polydactyly-type 4 (PPD4, OMIM: 174700),^8^ pathogenic variants in *GLI3* have been described. Out of these diseases, Pallister-Hall syndrome is the most distinct entity, defined by the presence of central polydactyly and hypothalamic hamartoma.^9^ The other *GLI3* syndromes are defined by the presence of preaxial and/or postaxial polydactyly of the hand and feet with or without syndactyly (Greig syndrome, PPD4). Also, various mild craniofacial features such as hypertelorism and macrocephaly can occur. Pallister-Hall syndrome is caused by truncating variants in the middle third of the *GLI3* gene.^10–12^ The truncation of GLI3 causes an overexpression of GLI3R, which is believed to be the key difference between Pallister-Hall and the GLI3*-*mediated polydactyly syndromes.^9 11^ Although multiple attempts have been made, the clinical and genetic distinction between the GLI3-mediated polydactyly syndromes is less evident. This has for example led to the introduction of subGreig and the formulation of an Oro-facial-digital overlap syndrome.^10^ Other authors, suggested that we should not regard these diseases as separate entities, but as a spectrum of GLI3-mediated polydactyly syndromes.^13^

Although phenotype/genotype correlation of the different syndromes has been cumbersome, clinical and animal studies do provide evidence that distinct regions within the gene, could be related to the individual anomalies contributing to these syndromes. First, case studies show isolated preaxial polydactyly is caused by both truncating and non-truncating variants throughout the *GLI3* gene, whereas in isolated postaxial polydactyly cases truncating variants at the C-terminal side of the gene are observed.^12 14^ These results suggest two different groups of variants for preaxial and postaxial polydactyly. Second, recent animal studies suggest that posterior malformations in GLI3-mediated polydactyly syndromes are likely related to a dosage effect of GLI3R rather than due to the influence of an altered GLI3A expression.^15^

Past attempts for phenotype/genotype correlation in GLI3-mediated polydactyly syndromes have directly related the diagnosed syndrome to the observed genotype.^10–12 16^ Focusing on individual hand phenotypes, such as preaxial and postaxial polydactyly and syndactyly might be more reliable because it prevents misclassification due to inconsistent use of syndrome definition. Subsequently, latent class analysis (LCA) provides the possibility to relate a group of observed variables to a set of latent, or unmeasured, parameters and thereby identifying different subgroups in the obtained dataset.^17^ As a result, LCA allows us to group different phenotypes within the GLI3-mediated polydactyly syndromes and relate the most important predictors of the grouped phenotypes to the observed *GLI3* variants.

The aim of our study was to further investigate the correlation of the individual phenotypes to the genotypes observed in GLI3-mediated polydactyly syndromes, using LCA. Cases were obtained by both literature review and the inclusion of local clinical cases. Subsequently, we identified two subclasses of limb anomalies that relate to the underlying *GLI3* variant. We provide evidence for two different phenotypic and genotypic groups with predominantly preaxial and postaxial hand and feet anomalies, and we specify those cases with a higher risk for corpus callosum anomalies.

## Methods

### Literature review

The Human Gene Mutation Database (HGMD Professional 2019) was reviewed to identify known pathogenic variants in *GLI3* and corresponding phenotypes.^18^ All references were obtained and cases were included when they were diagnosed with either Greig or subGreig syndrome or PPD4.^10–12^ Pallister-Hall syndrome and acrocallosal syndrome were excluded because both are regarded distinct syndromes and rather defined by the presence of the non-hand anomalies, than the presence of preaxial or postaxial polydactyly.^13 19^ Isolated preaxial or postaxial polydactyly were excluded for two reasons: the phenotype/genotype correlations are better understood and both anomalies can occur sporadically which could introduce falsely assumed pathogenic *GLI3* variants in the analysis. Additionally, cases were excluded when case-specific phenotypic or genotypic information was not reported or if these two could not be related to each other. Families with a combined phenotypic description, not reducible to individual family members, were included as one case in the analysis.

### Clinical cases

The Sophia Children’s Hospital Database was reviewed for cases with a *GLI3* variant. Within this population, the same inclusion criteria for the phenotype were valid. Relatives of the index patients were also contacted for participation in this study, when they showed comparable hand, foot, or craniofacial malformations or when a *GLI3* variant was identified. Phenotypes of the hand, foot and craniofacial anomalies of the patients treated in the Sophia Children's Hospital were collected using patient documentation. Family members were identified and if possible, clinically verified. Alternatively, family members were contacted to verify their phenotypes. If no verification was possible, cases were excluded.

### Phenotypes

The phenotypes of both literature cases and local cases were extracted in a similar fashion. The most frequently reported limb and craniofacial phenotypes were dichotomised. The dichotomised hand and foot phenotypes were preaxial polydactyly, postaxial polydactyly and syndactyly. Broad halluces or thumbs were commonly reported by authors and were dichotomised as a presentation of preaxial polydactyly. The extracted dichotomised craniofacial phenotypes were hypertelorism, macrocephaly and corpus callosum agenesis. All other phenotypes were registered, but not dichotomised.

### Pathogenic GLI3 variants

All *GLI3* variants were extracted and checked using Alamut Visual V.2.14. If indicated, variants were renamed according to standard Human Genome Variation Society nomenclature.^20^ Variants were grouped in either missense, frameshift, nonsense or splice site variants. In the group of frameshift variants, a subgroup with possible splice site effect were identified for subgroup analysis when indicated. Similarly, nonsense variants prone for nonsense mediated decay (NMD) and nonsense variants with experimentally confirmed NMD were identified.^21^ Deletions of multiple exons, CNVs and translocations were excluded for analysis. A full list of included mutations is available in the online supplementary materials.

10.1136/jmedgenet-2020-106948.supp1 Supplementary data

**Figure 1:**
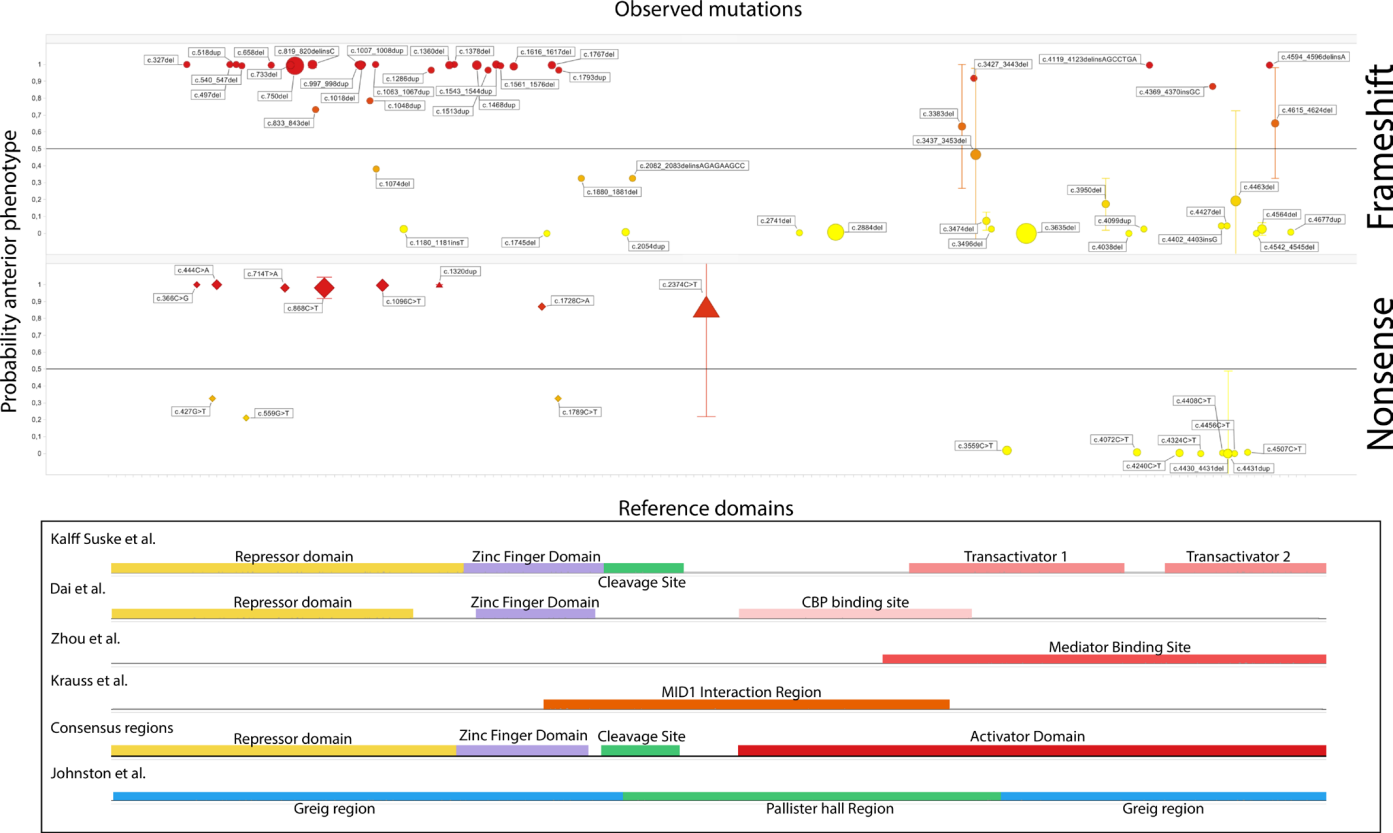
In this figure the posterior probability of an anterior phenotype is plotted against the location of the variant, stratified for the type of mutation that was observed. For better overview, only variants with a location effect were displayed. The full figure, including all variant types, can be found in the online supplementary figure 1. Each mutation is depicted as a dot, the size of the dot represents the number of observations for that variant. If multiple observations were made, the mean posterior odds and IQR are plotted. For the nonsense variants, variants that were predicted to produce nonsense mediated decay, are depicted using a triangle. Again, the size indicates the number of observations.

The location of the variant was compared with five known structural domains of the *GLI3* gene: (1) repressor domain, (2) zinc finger domain, (3) cleavage site, (4) activator domain, which we defined as a concatenation of the separately identified transactivation zones, the CBP binding domain and the mediator binding domain (MBD) and (5) the MID1 interaction region domain.^1 6 22–24^ The boundaries of each of the domains were based on available literature (figure 1, exact locations available in the online supplementary materials). The boundaries used by different authors did vary, therefore a consensus was made.

10.1136/jmedgenet-2020-106948.supp2 Supplementary data

### Latent class analysis

To cluster phenotypes and relate those to the genotypes of the patients, an explorative analysis was done using LCA in R (R V.3.6.1 for Mac; polytomous variable LCA, poLCA V.1.4.1.). We used our LCA to detect the number of phenotypic subgroups in the dataset and subsequently predict a class membership for each case in the dataset based on the posterior probabilities.

In order to make a reliable prediction, only phenotypes that were sufficiently reported and/or ruled out were feasible for LCA, limiting the analysis to preaxial polydactyly, postaxial polydactyly and syndactyly of the hands and feet. Only full cases were included. To determine the optimal number of classes, we fitted a series of models ranging from a one-class to a six-class model. The optimal number of classes was based on the conditional Akaike information criterion (cAIC), the non adjusted and the sample-size adjusted Bayesian information criterion (BIC and aBIC) and the obtained entropy.^25^ The explorative LCA produces both posterior probabilities per case for both classes and predicted class membership. Using the predicted class membership, the phenotypic features per class were determined in a univariate analysis (χ^2^, SPSS V.25). Using the posterior probabilities on latent class (LC) membership, a scatter plot was created using the location of the variant on the x-axis and the probability of class membership on the y-axis for each of the types of variants (Tibco Spotfire V.7.14). Using these scatter plots, variants that give similar phenotypes were clustered.

### Genotype/phenotype correlation

Because an LC has no clinical value, the correlation between genotypes and phenotypes was investigated using the predictor phenotypes and the clustered phenotypes. First, those phenotypes that contribute most to LC membership were identified. Second those phenotypes were directly related to the different types of variants (missense, nonsense, frameshift, splice site) and their clustered locations. Quantification of the relation was performed using a univariate analysis using a χ^2^ test. Because of our selection criteria, meaning patients at least have two phenotypes, a multivariate using a logistic regression analysis was used to detect the most significant predictors in the overall phenotype (SPSS V.25). Finally, we explored the relation of the clustered genotypes to the presence of corpus callosum agenesis, a rare malformation in GLI3-mediated polydactyly syndromes which cannot be readily diagnosed without additional imaging.

## Results

We included 251 patients from the literature and 46 local patients,^10–12 16 21 26–43^ in total 297 patients from 155 different families with 127 different *GLI3* variants, 32 of which were large deletions, CNVs or translocations. In six local cases, the exact variant could not be retrieved by status research.

The distribution of the most frequently observed phenotypes and variants are presented in table 1. Other recurring phenotypes included developmental delay (n=22), broad nasal root (n=23), frontal bossing or prominent forehead (n=16) and craniosynostosis (n=13), camptodactyly (n=8) and a broad first interdigital webspace of the foot (n=6).

**Table 1:** Baseline phenotypes and genotypes of selected population

Phenotypes	Affected/reported cases (n)	
Hand	Preaxial polydactyly	124/294
	Postaxial polydactyly	170/292
	Syndactyly	124/297
Foot	Preaxial polydactyly	238/297
	Postaxial polydactyly	70/295
	Syndactyly	193/297
Cranium	Macrocephaly	85/228
	Hypertelorism	92/237
	Corpus callosum	16/145
Genotypes		Cases (n)
Included in analysis	Frameshift	107
	Nonsense	68
	Missense	60
	Splice	24
Excluded in analysis	CNV	29
	Translocation	3
	No specific information on mutation	6

The LCA model was fitted using the six defined hand/foot phenotypes. Model fit indices for the LCA are displayed in table 2. Based on the BIC, a two-class model has the best fit for our data. The four-class model does show a gain in entropy, however with a higher BIC and loss of df. Therefore, based on the majority of performance statistics and the interpretability of the model, a two-class model was chosen. Table 3 displays the distribution of phenotypes and genotypes over the two classes.

**Table 2:** Model fit indices for the one-class through six-class model evaluated in our LCA

Number of classes	Log-likelihood	Residual df	BIC	aBIC	cAIC	Likelihood ratio	Entropy
1	−1072.0687	57	2178.316	2159.109	2184.316	299.59038	–
2	−966.4844	50	2006.632	1965.407	2019.632	88.42178	0.765
3	−949.9799	43	2013.288	1949.865	2033.288	55.41278	0.740
4	−942.9999	36	2038.993	1953.372	2065.993	41.45279	0.952
5	−937.2077	29	2067.074	1959.255	2101.074	29.86850	0.569
6	−933.5159	22	2099.355	1969.338	2140.355	22.48488	0.716

BIC, Bayesian information criterion; LCA, latent class analysis.

**Table 3:** Distribution of phenotypes and genotypes in the two latent classes (LC)

		LC 1/posterior phenotype	LC 2/anterior phenotype
Cases in LC (n)		88	201
Mean probability of class membership		0.91 (0.88–0.94)	0.96 (0.95–0.97)
Phenotypes		% of cases in class	
Hand	Preaxial polydactyly	15.91%	52.74%*
	Postaxial polydactyly	96.59%	40.80%*
	Syndactyly	12.50%	53.73%*
Foot	Preaxial polydactyly	45.45%	95.52%*
	Postaxial polydactyly	69.32%	1.49%*
	Syndactyly	23.86%	83.08%*
Cranium	Macrocephaly	29/60	54/162
	Hypertelorism	23/56	68/177
	Corpus callosum	8/44	8/98
Genotypes	Cases (n)		
	Total	85/88	173/201
Included mutations	Frameshift	52	54
	Nonsense	26	42
	Missense	6	54*
	Splice	1	23*

*P<0.00.

Table 1 depicts the baseline phenotypes and genotypes in the obtained population. Note incomplete data especially in the cranium phenotypes. In total 259 valid genotypes were present. In total, 289 cases had complete data for all hand and foot phenotypes (preaxial polydactyly, postaxial polydactyly and syndactyly) and thus were available for LCA. Combined, for phenotype/genotype correlation 258 cases were available with complete genotypes and complete hand and foot phenotypes.

Table 2 depicts the model fit indices for all models that have been fitted to our data.

Table 3 depicts the distribution of phenotypes and genotypes over the two assigned LCs. Hand and foot phenotypes were used as input for the LCA, thus are all complete cases. Malformation of the cranium and genotypes do have missing cases. Note that for the LCA, full case description was required, resulting in eight cases due to incomplete phenotypes. Out of these eight, one also had a genotype that thus needed to be excluded. Missingness of genotypic data was higher in LC2, mostly due to CNVs (table 1).

In 54/60 cases, a missense variant produced a posterior phenotype. Likewise, splice site variants show the same phenotype in 23/24 cases (table 3). For both frameshift and nonsense variants, this relation is not significant (52 anterior vs 54 posterior and 26 anterior vs 42 posterior, respectively). Therefore, only for nonsense and frameshift variants the location of the variant was plotted against the probability for LC2 membership in figure 1. A full scatterplot of all variants is available in online supplementary figure 1.

Figure 1 reveals a pattern for these nonsense and frameshift variants that reveals that variants at the C-terminal of the gene predict anterior phenotypes. When relating the domains of the GLI3 protein to the observed phenotype, we observe that the majority of patients with a nonsense or frameshift variant in the repressor domain, the zinc finger domain or the cleavage site had a high probability of an LC2/anterior phenotype. This group contains all variants that are either experimentally determined to be subject to NMD (triangle marker in figure 1) or predicted to be subject to NMD (diamond marker in figure 1). Frameshift and nonsense variants in the activator domain result in high probability for an LC1/posterior phenotype. These variants will be further referred to as truncating variants in the activator domain.

The univariate relation of the individual phenotypes to these two groups of variants are estimated and presented in table 4. In our multivariate analysis, postaxial polydactyly of the foot and hand are the strongest predictors (Beta: 2.548, p<0001 and Beta: 1.47, p=0.013, respectively) for patients to have a truncating variant in the activator domain. Moreover, the effect sizes of preaxial polydactyly of the hand and feet (Beta: −0.797, p=0123 and −1.772, p=0.001) reveals that especially postaxial polydactyly of the foot is the dominant predictor for the genetic substrate of the observed anomalies.

**Table 4:** Univariate and multivariate analysis of the phenotype/genotype correlation

			Univariate analysis	Multivariate analysis	
			OR frameshift/nonsense mutation 5′ side of the zinc finger domain	Beta	P value
Phenotype	Hand	Preaxial polydactyly	0.27 (CI: 0.14 – 0.54)	−0.797	0.123
		Postaxial polydactyly	12.7 (CI: 5.2 – 31.0)	1.469	0.013
		Syndactyly	0.3 (CI: 0.16 – 0.57)	0.505	0.338
	Foot	Preaxial polydactyly	0.1 (CI: 0.032 – 0.14)	−1.772	0.001
		Postaxial polydactyly	33.9 (CI: 15.1 – 76.0)	2.548	<0.001
		Syndactyly	0.1 (CI: 0.054 – 0.19)	−1.773	<0.001
Regression constant				−0.564	0.729

Table 4 shows exploration of the individual phenotypes on the genotype, both univariate and multivariate. The multivariate analysis corrects for the presence of multiple phenotypes in the underlying population.

Although the craniofacial anomalies could not be included in the LCA, the relation between the observed anomalies and the identified genetic substrates can be studied. The prevalence of hypertelorism was equally distributed over the two groups of variants (47/135 vs 21/47 respectively, p<0.229). However for corpus callosum agenesis and macrocephaly, there was a higher prevalence in patients with a truncating variant in the activator domain (3/75 vs 11/41, p<0.001; OR: 8.8, p<0.001) and 42/123 vs 24/48, p<0.05). Noteworthy is the fact that 11/14 cases with corpus callosum agenesis in the dataset had a truncating variant in the activator domain.

## Discussion

In this report, we present new insights into the correlation between the phenotype and the genotype in patients with GLI3-mediated polydactyly syndromes. We illustrate that there are two LCs of patients, best predicted by postaxial polydactyly of the hand and foot for LC1, and the preaxial polydactyly of the hand and foot and syndactyly of the foot for LC2. Patients with postaxial phenotypes have a higher risk of having a truncating variant in the activator domain of the *GLI3* gene which is also related to a higher risk of corpus callosum agenesis. These results suggest a functional difference between truncating variants on the N-terminal and the C-terminal side of the GLI3 cleavage site.

Previous attempts of phenotype to genotype correlation have not yet provided the clinical confirmation of these assumed mechanisms in the pathophysiology of GLI3-mediated polydactyly syndromes. Johnston *et al* have successfully determined the Pallister-Hall region in which truncating variants produce a Pallister-Hall phenotype rather than Greig syndrome.^11^ However, in their latest population study, subtypes of both syndromes were included to explain the full spectrum of observed malformations. In 2015, Demurger *et al* reported the higher incidence of corpus callosum agenesis in the Greig syndrome population with truncating mutations in the activator domain.^12^ Al-Qattan in his review summarises the concept of a spectrum of anomalies dependent on haplo-insufficiency (through different mechanisms) and repressor overexpression.^13^ However, he bases this theory mainly on reviewed experimental data. Our report is the first to provide an extensive clinical review of cases that substantiate the phenotypic difference between the two groups that could fit the suggested mechanisms. We agree with Al-Qattan *et al* that a variation of anomalies can be observed given any pathogenic variant in the GLI3 gene, but overall two dominant phenotypes are present: a population with predominantly preaxial anomalies and one with postaxial anomalies. The presence of preaxial or postaxial polydactyly and syndactyly is not mutually exclusive for one of these two subclasses; meaning that preaxial polydactyly can co-occur with postaxial polydactyly. However, truncating mutations in the activator domain produce a postaxial phenotype, as can be derived from the risk in table 4. The higher risk of corpus callosum agenesis in this population shows that differentiating between a preaxial phenotype and a postaxial phenotype, instead of between the different GLI3-mediated polydactyly syndromes, might be more relevant regarding diagnostics for corpus callosum agenesis.

We chose to use LCA as an exploratory tool only in our population for two reasons. First of all, LCA can be useful to identify subgroups, but there is no ‘true’ model or number of subgroups you can detect. The best fitting model can only be estimated based on the available measures and approximates the true subgroups that might be present. Second, LC membership assignment is a statistical procedure based on the posterior probability, with concordant errors of the estimation, rather than a clinical value that can be measured or evaluated. Therefore, we decided to use our LCA only in an exploratory tool, and perform our statistics using the actual phenotypes that predict LC membership and the associated genotypes. Overall, this method worked well to differentiate the two subgroups present in our dataset. However, outliers were observed. A qualitative analysis of these outliers is available in the online supplementary data.

The genetic substrate for the two phenotypic clusters can be discussed based on multiple experiments. Overall, we hypothesise two genetic clusters: one that is due to haploinsufficiency and one that is due to abnormal truncation of the activator. The hypothesised cluster of variants that produce haploinsufficiency is mainly based on the experimental data that confirms NMD in two variants and the NMD prediction of other nonsense variants in Alamut. For the frameshift variants, it is also likely that the cleavage of the zinc finger domain results in functional haploinsufficiency either because of a lack of signalling domains or similarly due to NMD. Missense variants could cause haploinsufficiency through the suggested mechanism by Krauss *et al* who have illustrated that missense variants in the MID1 domain hamper the functional interaction with the MID1-α4-PP2A complex, leading to a subcellular location of GLI3.^24^ The observed missense variants in our study exceed the region to which Krauss *et al* have limited the MID-1 interaction domain. An alternative theory is suggested by Zhou *et al* who have shown that missense variants in the MBD can cause deficiency in the signalling of GLI3A, functionally implicating a relative overexpression of GLI3R.^22^ However, GLI3R overexpression would likely produce a posterior phenotype, as determined by Hill *et al* in their fixed homo and hemizygous GLI3R models.^15^ Therefore, our hypothesis is that all included missense variants have a similar pathogenesis which is more likely in concordance with the mechanism introduced by Krauss *et al*. To our knowledge, no splice site variants have been functionally described in literature. However, it is noted that the 15 and last exon encompasses the entire activator domain, thus any splice site mutation is by definition located on the 5′ side of the activator. Based on the phenotype, we would suggest that these variants fail to produce a functional protein. We hypothesise that the truncating variants of the activator domain lead to overexpression of GLI3R in SHH rich areas. In normal development, the presence of SHH prevents the processing of full length GLI3^4^ into GLI3R, thus producing the full length activator. In patients with a truncating variant of the activator domain of GLI3, thus these variants likely have the largest effect in SHH rich areas, such as the ZPA located at the posterior side of the hand/footplate. Moreover, the lack of posterior anomalies in the GLI3^∆699/-^ mouse model (hemizygous fixed repressor model) compared with the GLI3^∆699/∆699^ mouse model (homozygous fixed repressor model), suggesting a dosage effect of GLI3R to be responsible for posterior hand anomalies.^15^ These findings are supported by Lewandowski *et al*, who show that the majority of the target genes in GLI signalling are regulated by GLI3R rather than GLI3A.^44^ Together, these findings suggest a role for the location and type of variant in GLI3-mediated syndromes.

Interestingly, the difference between Pallister-Hall syndrome and GLI3-mediated polydactyly syndromes has also been attributed to the GLI3R overexpression. However, the difference in phenotype observed in the cases with a truncating variant in the activator domain and Pallister-Hall syndrome suggest different functional consequences. When studying figure 1, it is noted that the included truncating variants on the 3′ side of the cleavage site seldomly affect the CBP binding region, which could provide an explanation for the observed differences. This binding region is included in the Pallister-Hall region as defined by Johnston *et al* and is necessary for the downstream signalling with GLI1.^10 11 23 45^ Interestingly, recent reports show that pathogenic variants in GLI1 can produce phenotypes concordant with Ellis von Krefeld syndrome, which includes overlapping features with Pallister-Hall syndrome.^46^ The four truncating variants observed in this study that do affect the CBP but did not result in a Pallister-Hall phenotype are conflicting with this theory. Krauss *et al* postulate an alternative hypothesis, they state that the MID1-α4-PP2A complex, which is essential for GLI3A signalling, could also be the reason for overlapping features of Opitz syndrome, caused by variants in MID1, and Pallister-Hall syndrome. Further analysis is required to fully appreciate the functional differences between truncating mutations that cause Pallister-Hall syndrome and those that result in GLI3-mediated polydactyly syndromes.

For the clinical evaluation of patients with GLI3-mediated polydactyly syndromes, intracranial anomalies are likely the most important to predict based on the variant. Unfortunately, the presence of corpus callosum agenesis was not routinely investigated or reported thus this feature could not be used as an indicator phenotype for LC membership. Interestingly when using only hand and foot phenotypes, we did notice a higher prevalence of corpus callosum agenesis in patients with posterior phenotypes. The suggested relation between truncating mutations in the activator domain causing these posterior phenotypes and corpus callosum agenesis was statistically confirmed (OR: 8.8, p<0.001). Functionally this relation could be caused by the GLI3-MED12 interaction at the MBD: pathogenic DNA variants in MED12 can cause Opitz-Kaveggia syndrome, a syndrome in which presentation includes corpus callosum agenesis, broad halluces and thumbs.^47^

In conclusion, there are two distinct phenotypes within the GLI3-mediated polydactyly population: patients with more posteriorly and more anteriorly oriented hand anomalies. Furthermore, this difference is related to the observed variant in GLI3. We hypothesise that variants that cause haploinsufficiency produce anterior anomalies of the hand, whereas variants with abnormal truncation of the activator domain have more posterior anomalies. Furthermore, patients that have a variant that produces abnormal truncation of the activator domain, have a greater risk for corpus callosum agenesis. Thus, we advocate to differentiate preaxial or postaxial oriented *GLI3* phenotypes to explain the pathophysiology as well as to get a risk assessment for corpus callosum agenesis.

## Data Availability

Data are available upon reasonable request.
