# Supplementary material for: Variant type and position predict two distinct limb phenotypes in patients with GLI3-mediated polydactyly syndromes
Source: J Med Genet. 2020 Jun 26;58(6):362–8. doi: 10.1136/jmedgenet-2020-106948 (PMC8142428; doi:10.1136/jmedgenet-2020-106948)
Supplement: Supplementary data [file jmedgenet-2020-106948supp001.pdf]

Supplementary materials

1. Structural domains *GLI3* gene

Author	Domain	Amino acids
Kalf-Susske et al.[1]	Repressor	1-462
	Zinc Finger Domain	462-645
	Proteolytic Cleavage site	645-748
	TA1	1376-1580
	TA2	1044-1322
Dai et al.[2]	Repressor	1-396
	Zinc Finger domain	480-636
	CBP binding site	826-1132
Johnston et al[3-5]	Pallister Hall region	667-1160
Kraus et al. [6]	Repressor	1-397
	Zinc Finger domain	480-632
	Cleavage site	650-750
	Activator domain	827-1132
	MID1-interaction region	568-1100
Zhou et al.[7]	Mediator binding domain	1006-1596

2. Included variants in the analysis

Variant	Protein	Type	Observations	Median probability LC2
c.327del	p.Phe109Leufs*50	frameshift	1	0,999
c.497del	p.Pro166Leufs*50	frameshift	1	0,999
c.518dup	p.Ile174Hisfs*2	frameshift	1	0,999
c.540_547del	p.Asn181Cysfs*15	frameshift	1	0,994
c.658del	p.Arg220Valfs*3	frameshift	1	0,996
c.733del	p.Thr245Leufs*65	frameshift	2	0,997
c.750del	p.Tyr251Metfs*59	frameshift	11	0,967
c.819_820delinsC	p.Met274Trpfs*36	frameshift	3	0,999
c.833_843del	p.Arg278Thrfs*22	frameshift	1	0,733
c.997_998dup	p.Tyr334Profs*14	frameshift	1	1,000
c.1007_1008dup	p.Leu337Thrfs*11	frameshift	3	0,996
c.1018del	p.Ser340Valfs*7	frameshift	1	0,999
c.1048dup	p.Tyr350Leufs*62	frameshift	1	0,786
c.1063_1067dup	p.Leu357Serfs*10	frameshift	1	1,000
c.1074del	p.His358Glnfs*7	frameshift	1	0,383
c.1180_1181insT	p.Pro394Leufs*18	frameshift	2	0,025
c.1286dup	p.Met430Aspfs*12	frameshift	1	0,967
c.1360del	p.Gln454Serfs*48	frameshift	2	0,997
c.1378del	p.Val461Serfs*41	frameshift	1	1,000
c.1468dup	p.Glu490Glyfs*14	frameshift	3	0,996
c.1513dup	p.His505Profs*47	frameshift	1	0,967
c.1543_1544dup	p.Arg516Alafs*20	frameshift	2	0,999
c.1561_1576del	p.Ser521Profs*9	frameshift	1	0,994
c.1616_1617del	p.Arg539Thrfs*12	frameshift	1	0,996
c.1617_1633del	p.Arg539Serfs*7	frameshift	1	0,981
c.1745del	p.Gly582Valfs*47	frameshift	1	0,000
c.1767del	p.Asn589Lysfs*40	frameshift	2	0,997
c.1793dup	p.Asn598Lysfs*7	frameshift	1	0,967
c.1880_1881del	p.His627Argfs*48	frameshift	1	0,326
c.2054dup	p.Arg686Alafs*52	frameshift	2	0,006
c.2082_2083delinsAGAGAAGCC	p.Val695Glufs*45	frameshift	1	0,326
c.2741del	p.Gly914Alafs*38	frameshift	1	0,003
c.2884del	p.Asp962Metfs*41	frameshift	9	0,019
c.3383del	p.Asp1128Alafs*78	frameshift	2	0,632
c.3427_3443del	p.Phe1143Alafs*98	frameshift	1	0,919
c.3437_3453del	p.Leu1146Argfs*95	frameshift	4	0,466
c.3474del	p.Ile1160Phefs*46	frameshift	2	0,073
c.3496del	p.Ser1166Alafs*40	frameshift	1	0,024
c.3635del	p.Gly1212Alafs*18	frameshift	14*	0,005
c.3950del	p.Pro1317Glnfs*102	frameshift	2	0,173
c.4038del	p.Gln1347Argfs*72	frameshift	1	0,001

c.4099dup	p.Ala1367Glyfs*45	frameshift	1	0,024
c.4119_4123delinsAGCCTGA	p.Pro1374Alafs*2	frameshift	1	0,996
c.4369_4370insGC	p.Ala1457Glyfs*32	frameshift	1	0,870
c.4402_4403insG	p.Leu1468Argfs*11	frameshift	1	0,043
c.4427del	p.Asn1476Thrfs*12	frameshift	1	0,043
c.4463del	p.Thr1488Lysfs*23	frameshift	4	0,355
c.4542_4545del	p.His1515Profs*3	frameshift	1	0,001
c.4564del	p.Ala1522Profs*2	frameshift	3	0,025
c.4594_4596delinsA	p.Ser1532Thrfs*2	frameshift	1	0,996
c.4615_4624del	p.Thr1539Glyfs*11	frameshift	2	0,654
c.4677dup	p.Gly1560Argfs*38	frameshift	1	0,006
c.1446C>G	p.Cys482Trp	missense	2	0,800
c.1498C>T	p.His500Tyr	missense	3	0,999
c.1559G>A	p.Cys520Tyr	missense	1	0,979
c.1627G>A	p.Glu543Lys	missense	3	0,211
c.1633C>A	p.Pro545Thr	missense	3	0,999
c.1658G>A	p.Cys553Tyr	missense	1	0,870
c.1733G>C	p.Cys578Ser	missense	1	0,996
c.1748G>T	p.Cys583Phe	missense	1	0,870
c.1786C>T	p.His596Tyr	missense	3	0,919
c.1787A>C	p.His596Pro	missense	2	0,999
c.1826G>A	p.Cys609Tyr	missense	11	0,019
c.1873C>T	p.Arg625Trp	missense	7	0,967
c.1874G>A	p.Arg625Gln	missense	4	0,994
c.2686G>A	p.Asp896Asn	missense	1	0,999
c.2690C>G	p.Pro897Arg	missense	6	0,996
c.2708C>T	p.Ser903Leu	missense	4	0,994
c.2721C>G	p.Ser907Arg	missense	2	0,997
c.3018C>A	p.Ser1006Arg	missense	4	0,984
c.3534G>C	p.Lys1178Asn	missense	1	0,980
c.366C>G	p.Tyr122*	nonsense	1	0,999
c.427G>T	p.Glu143*	nonsense	1	0,326
c.444C>A	p.Tyr148*	nonsense	4	0,990
c.559G>T	p.Glu187*	nonsense	1	0,211
c.714T>A	p.Tyr238*	nonsense	3	0,980
c.868C>T	p.Arg290*	nonsense	13	0,981
c.1096C>T	p.Arg366*	nonsense	6	0,997
c.1320dup	p.Glu441*	nonsense	1	0,999
c.1728C>A	p.Tyr576*	nonsense	2	0,870
c.1789C>T	p.Gln597*	nonsense	1	0,326
c.2374C>T	p.Arg792*	nonsense	18	0,895
c.3559C>T	p.Gln1187*	nonsense	3	0,019
c.4072C>T	p.Gln1358*	nonsense	2	0,005
c.4240C>T	p.Gln1414*	nonsense	2	0,003
c.4324C>T	p.Gln1442*	nonsense	1	0,001

c.4408C>T	p.Gln1470*	nonsense	1	0,003
c.4430_4431del	p.Ser1477*	nonsense	3	0,000
c.4431dup	p.Glu1478*	nonsense	2	0,000
c.4432G>T	p.Glu1478*	nonsense	1	0,981
c.4456C>T	p.Gln1486*	nonsense	1	0,000
c.4507C>T	p.Gln1503*	nonsense	1	0,006
c.474-2A>G	p.?	splice	5	1,000
c.679+1G>T	p.?	splice	1	0,682
c.679+2_679+15del	p.?	splice	3	0,999
c.827-3C>G	p.?	splice	2	0,434
c.1497+1G>C	p.?	splice	1	0,870
c.1497+1G>A	p.?	splice	2	0,800
c.1497+1G>T	p.?	splice	2	0,987
c.1497+2T>G	p.?	splice	3	0,967
c.1498-1G>C	p.?	splice	3	0,996
c.1647+2_1647+3del	p.?	splice	2	1,000

*One case misses complete phenotypic discription

3. Qualitative analysis of outliers in the phenotype/genotype correlation:

Overall the distinction of genotypes based on phenotypes is well defined, however outliers in our analysis were present. Four outliers were identified in the group of truncating variants in the N-terminal side of the gene: c.427 G>T(p.Glu143*), c.559 G>T(p.Glu187*), c.1789C>T(p.Gln597*) and c.2374C>T(p.Arg792*) (Figure 1). Strikingly, the c.2374C>T(p.Arg792*) variant has been experimentally confirmed to produce NMD but produced a variable phenotype. In the case review of patients with this variant, the consensus phenotype of this variant is postaxial polydactyly of the hand, preaxial polydactyly of the foot and syndactyly, thus concordant with the rest of the haploinsufficiency variants. Looking at the effect measures in our regression analysis (Beta's +1,47, -1,77 and -1,77 respectively), this is rightfully classified a preaxial phenotype. The majority of frameshift variants on the 5' side of the cleavage site produced a preaxial phenotype, 3 outliers were observed: c.1074del(p.His358Glnfs*7), c.1180_1181insT(p.Pro394Leufs*18), c.1745del(p.Gly582Valfs*47). The c.1074del(p.His358Glnfs*7) variant was included as a single phenotypic description by the original authors although the variant was present in a larger pedigree. Thus the penetrance of e.g. postaxial polydactyly is unknown but could strongly affect the prediction. The c.1745del(p.Gly582Valfs*47) variant produced a true postaxial phenotype, this variant is located in the zinc finger domain. We hypothesize that the unaffected part of this domain could maintain some function in the produced protein. Frameshift variants on the 3' side of the zinc finger domain, more variability on the phenotype was observed: c.3383del(p.Asp1128Alafs*78), c.3427_3443del(p.Phe1143Alafs*98) ,c.3437_3453del(p.Leu1146Argfs*95), c.4119_4123delinsAGCCTGA(p.Pro1374Alafs*2), c.4369_4370insGC(p.Ala1457Glyfs*32), c.4594_4596delinsA(p.Ser1532Thrfs*2) and c.4615_4624del(p.Thr1539Glyfs*11) all showed a variable or preaxial dominant phenotype. The deletion of multiple nucleotides for most of these variants is noted, however no exact

mechanism is apparent for the difference in phenotypic presentation. Alternative splicing could explain the preaxial phenotype, however was not predicted in Alamut. There was 1 missense variant with increased prevalence of postaxial polydactyly, on individual review these were the c.1627G>A(p.Glu543Lys) variants observed in our clinic. This local variant was classified as a variant of unknown significance according to the ACMG guidelines and was observed in all 3 tested cases. Two more family members are symptomatic, but were not tested for this variant. We chose to exclude these 2 unconfirmed cases due to the uncertain pathogenicity of the variant. Nevertheless it is noteworthy that the excluded cases had a preaxial phenotype. Moreover, the single case with a full anterior phenotype did have abducted, but normally sized, halluces. Further confirmation of this variant is required to confirm its pathogenicity and phenotype.

4. Excluded variants

There are a number of variants not included in our analysis that have not been discussed in the manuscript, namely the variants that produced isolated hand or feet phenotypes. Supplementary figure 1 reveals that the included missense variants center around the MID1 interaction region. However, when reviewing the HGMD database, more missense variants are present on the N and C terminal side of the gene. These variants cause isolated preaxial polydactyly and postaxial polydactyly[1,8-11], but also atrial septal defects, urinary tract anomalies, esophageal atresia and medulloblastoma have been described[12-14]. Missense variants in the N-terminal side of the gene likely produce a non-functional repressor with a functional activator. Since GLI3A seems to have no separate role in the etiology of polydactyly (especially on the posterior side), the hand phenotype is indeed expected to be comparable to haploinsufficiency. On the other hand, C-terminal missense variants likely hamper the downstage signaling of the activator as suggested by Zhou et al., which as discussed in the manuscript leads to relative repressor overexpression.

5. References used in supplementary materials

1. Kalff-Suske M, Wild A, Topp J, Wessling M, Jacobsen EM, Bornholdt D, Engel H, Heuer H, Aalfs CM, Ausems MG, Barone R, Herzog A, Heutink P, Homfray T, Gillessen-Kaesbach G, Konig R, Kunze J, Meinecke P, Muller D, Rizzo R, Strenge S, Superti-Furga A, Grzeschik KH. Point mutations throughout the GLI3 gene cause Greig cephalopolysyndactyly syndrome. *Hum Mol Genet.* 1999;8(9):1769-1777.
2. Dai P, Akimaru H, Tanaka Y, Maekawa T, Nakafuku M, Ishii S. Sonic Hedgehog-induced activation of the Gli1 promoter is mediated by GLI3. *J Biol Chem.* 1999;274(12):8143-8152.
3. Johnston JJ, Olivos-Glander I, Turner J, Aleck K, Bird LM, Mehta L, Schimke RN, Heilstedt H, Spence JE, Blancato J, Biesecker LG. Clinical and molecular delineation of the Greig cephalopolysyndactyly contiguous gene deletion syndrome and its distinction from acrocallosal syndrome. *Am J Med Genet A.* 2003;123A(3):236-242.
4. Johnston JJ, Olivos-Glander I, Killoran C, Elson E, Turner JT, Peters KF, Abbott MH, Aughton DJ, Aylsworth AS, Bamshad MJ, Booth C, Curry CJ, David A, Dinulos MB, Flannery DB, Fox MA, Graham JM, Grange DK, Guttmacher AE, Hannibal MC, Henn W, Hennekam RC, Holmes LB, Hoyme HE, Leppig KA, Lin AE, Macleod P, Manchester DK, Marcelis C, Mazzanti L, McCann E, McDonald MT, Mendelsohn NJ, Moeschler JB, Moghaddam B, Neri G, Newbury-Ecob R, Pagon RA, Phillips JA, Sadler LS, Stoler JM, Tilstra D, Walsh Vockley CM, Zackai EH, Zadeh TM, Brueton L, Black GC, Biesecker LG. Molecular and clinical analyses of Greig cephalopolysyndactyly and Pallister-Hall syndromes: robust phenotype prediction from the type and position of GLI3 mutations. *Am J Hum Genet.* 2005;76(4):609-622.
5. Johnston JJ, Sapp JC, Turner JT, Amor D, Aftimos S, Aleck KA, Bocian M, Bodurtha JN, Cox GF, Curry CJ, Day R, Donnai D, Field M, Fujiwara I, Gabbett M, Gal M, Graham JM, Hedera P, Hennekam RC, Hersh JH, Hopkin RJ, Kayserili H, Kidd AM, Kimonis V, Lin AE, Lynch SA, Maisenbacher M, Mansour S, McGaughran J, Mehta L, Murphy H, Raygada M, Robin NH, Rope AF, Rosenbaum KN, Schaefer GB, Shealy A, Smith W, Soller M, Sommer A, Stalker HJ, Steiner B, Stephan MJ, Tilstra D, Tomkins S, Trapane P, Tsai AC, Van Allen MI, Vasudevan PC, Zabel B, Zunich J, Black GC, Biesecker LG. Molecular analysis expands the spectrum of phenotypes associated with GLI3 mutations. *Hum Mutat.* 2010;31(10):1142-1154.
6. Krauss S, So J, Hambrock M, Kohler A, Kunath M, Scharff C, Wessling M, Grzeschik KH, Schneider R, Schweiger S. Point mutations in GLI3 lead to misregulation of its subcellular localization. *PloS one.* 2009;4(10):e7471.
7. Zhou H, Kim S, Ishii S, Boyer TG. Mediator modulates Gli3-dependent Sonic hedgehog signaling. *Mol Cell Biol.* 2006;26(23):8667-8682.
8. Demurger F, Ichkou A, Mougou-Zerelli S, Le Merrer M, Goudefroye G, Delezoide AL, Quelin C, Manouvrier S, Baujat G, Fradin M, Pasquier L, Megarbane A, Faivre L, Baumann C, Nampoothiri S, Roume J, Isidor B, Lacombe D, Delrue MA, Mercier S, Philip N, Schaefer E, Holder M, Krause A, Laffargue F, Sinico M, Amram D, Andre G, Liquier A, Rossi M, Amiel J, Giuliano F, Boute O, Dieux-Coeslier A, Jacquemont ML, Afenjar A, Van Maldergem L, Lackmy-Port-Lis M, Vincent-Delorme C, Chauvet ML, Cormier-Daire V, Devisme L, Genevieve D, Munnich A, Viot G, Raoul O, Romana S, Gonzales M, Encha-Razavi F, Odent S, Vekemans M, Attie-Bitach T. New insights into genotype-phenotype correlation for GLI3 mutations. *Eur J Hum Genet.* 2015;23(1):92-102.
9. Driess S, Freese K, Bornholdt D, Kobelt A, Kress W, Mortier G, Radhakrishna U, Antonarakis SE, Rauch A, Suri M, Verheij JB, Woerle H, Grzeschik KH, Kalff-Suske M. Gene symbol: GLI3. Disease: Greig cephalopolysyndactyly syndrome. *Hum Genet.* 2003;112(1):103.
10. Xiang Y, Wang Z, Bian J, Xu Y, Fu Q. Exome sequencing reveals a novel nonsense mutation of GLI3 in a Chinese family with 'non-syndromic' pre-axial polydactyly. *J Hum Genet.* 2016;61(10):907-910.

11. Rao C, Chen J, Peng Q, Mo Q, Xia X, Lu X. Mutational Screening of GLI3, SHH, and SHH ZRS in 78 Chinese Children with Nonsyndromic Polydactyly. *Genet Test Mol Biomarkers.* 2018;22(9):577-581.
12. Yang L, Shen C, Mei M, Zhan G, Zhao Y, Wang H, Huang G, Qiu Z, Lu W, Zhou W. De novo GLI3 mutation in esophageal atresia: reproducing the phenotypic spectrum of Gli3 defects in murine models. *Biochimica et biophysica acta.* 2014;1842(9):1755-1761.
13. Koole O, Houben RM, Mzembe T, Van Boeckel TP, Kayange M, Jahn A, Chimbwandira F, Glynn JR, Crampin AC. Improved retention of patients starting antiretroviral treatment in Karonga District, northern Malawi, 2005-2012. *J Acquir Immune Defic Syndr.* 2014;67(1):e27-e33.
14. Nicolaou N, Pulit SL, Nijman IJ, Monroe GR, Feitz WF, Schreuder MF, van Eerde AM, de Jong TP, Giltay JC, van der Zwaag B, Havenith MR, Zwakenberg S, van der Zanden LF, Poelmans G, Cornelissen EA, Lilien MR, Franke B, Roeleveld N, van Rooij IA, Cuppen E, Bongers EM, Giles RH, Knoers NV, Renkema KY. Prioritization and burden analysis of rare variants in 208 candidate genes suggest they do not play a major role in CAKUT. *Kidney Int.* 2016;89(2):476-486.
