# Supplementary figures and images for: Variant type and position predict two distinct limb phenotypes in patients with GLI3-mediated polydactyly syndromes
Source: J Med Genet. 2020 Jun 26;58(6):362–8. doi: 10.1136/jmedgenet-2020-106948 (PMC8142428; doi:10.1136/jmedgenet-2020-106948)

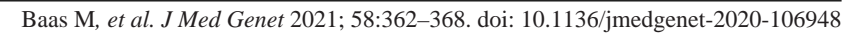

Supplement: Supplementary data [file jmedgenet-2020-106948supp002.pdf]
